# Supplementary material for: Reconciling Mining with the Conservation of Cave Biodiversity: A Quantitative Baseline to Help Establish Conservation Priorities
Source: PLoS One. 2016 Dec 20;11(12):e0168348. doi: 10.1371/journal.pone.0168348 (PMC5173368; doi:10.1371/journal.pone.0168348)
Supplement: S1 Dataset — (ZIP) [file pone.0168348.s002.zip › Taxa/Serra Norte/SN_2013/N5S/Lista N5S-55.pdf]

CAVIDADE N5S-0055

Classe	Ordem	Fam/Outros	Gên/Outros	Espécie	S-U	U-S	Zon.
Arachnida	Acari	(Parasitengonina)		sp.17		X	A
Arachnida	Acari	Eupodidae		sp.1		X	A
Arachnida	Acari	Ixodidae		jovem	X		E
Arachnida	Acari	Laelapidae	*Cosmolaelaps*	sp.1		X	A
Arachnida	Acari	Rhaphignatidae		sp.62	X		P
Arachnida	Acari	Trombiculidae		sp.39		X	A
Arachnida	Acari	Trombidiidae		sp.15	X		A
Arachnida	Amblypygi	Phrynidae	*Heterophrynus*	*longicornis*		1	P
Arachnida	Araneae	Araneidae	*Alpaida*	aff. *negro*		X	E
Arachnida	Araneae	Barychelidae		jovem	1		E
Arachnida	Araneae	Caponiidae		sp.1	X	X	E/A
Arachnida	Araneae	Corinnidae	*Creugas*	sp.1		1	E
Arachnida	Araneae	Drymusidae	*Drymusa*	*spelunca*	X	X	E
Arachnida	Araneae	Ochyroceratidae	*Ochyrocera*	sp.3	X	X	E/A
Arachnida	Araneae	Ochyroceratidae	*Speocera*	sp.1		X	A
Arachnida	Araneae	Pholcidae	*Leptopholcus*	sp.1	X	X	E
Arachnida	Araneae	Salticidae		jovem	X		E
Arachnida	Araneae	Theraphosidae		jovem	3	1	E
Arachnida	Araneae	Theridiidae	*Dipoena*	sp.1		X	E
Arachnida	Araneae	Theridiidae	*Theridion*	sp.1	X		E
Arachnida	Araneae	Theridiosomatidae	*Plato*	sp.1	X	X	E
Arachnida	Opiliones	Sclerosomatidae	*Prionostema*	sp.1	1		E
Arachnida	Palpigradi	Eukoeneniidae	*Eukoenenia*	sp.1		X	A
Arachnida	Pseudoscorpiones	Bochicidae		sp.1	X	X	A
Arachnida	Pseudoscorpiones	Cheliferoidea		sp.1		X	A
Arachnida	Pseudoscorpiones	Chthoniidae	*Pseudochthonius*	sp.1		X	E
Arachnida	Scorpiones	Buthidae	*Ananteris*	sp.1		1	A
Chilopoda	Geophilomorpha	Schendylidae		sp.1		1	A
Chilopoda	Scolopendromorpha	Cryptopidae	*Cryptops*	jovem		3	E
Chilopoda	Scutigeromorpha	Pselliodidae	*Sphendononema*	*guildingii*	2		E
Diplopoda	Polydesmida	Chelodesmidae		jovem		1	E
Diplopoda	Polydesmida	Pyrgodesmidae		jovem	X	X	E/P/A
Diplopoda	Spirostreptida	Pseudonannolenidae	*Pseudonannolene*	jovem	2		E
Entognatha	Collembola	Entomobryidae		sp.2	X		E
Entognatha	Collembola	Entomobryidae		sp.4		X	E
Entognatha	Collembola	Isotomidae		sp.1	X	X	A
Entognatha	Collembola	Paronellidae		sp.1		X	E
Entognatha	Collembola	Sminthuroidea		sp.2	X	X	P/A
Entognatha	Diplura	Campodeidae		sp.1	X	X	E
Entognatha	Diplura	Projapygidae		sp.1	X		E
Insecta	Blattodea	Polyphagidae		sp.1	5	6	A
Insecta	Coleoptera	Staphylinidae		sp.22	X	X	E
Insecta	Diptera	Culicidae	*Culex*	sp.	X		E/P
Insecta	Diptera	Ephydridae		sp.		X	A
Insecta	Diptera	Phoridae	Metopininae	sp.	X		E
Insecta	Diptera	Psychodidae	Phlebotominae	sp.	X	X	E/P/A
Insecta	Heteroptera	Dipsocoroidea		sp.	X	X	E/A
Insecta	Heteroptera	Reduviidae	Emesinae	sp.		2	E
Insecta	Heteroptera	Reduviidae	Reduviinae	sp.	8	3	E/A

Insecta	Homoptera	Cixiidae		jovem		X	E
Insecta	Hymenoptera	Bethylidae		sp.2	X		E
Insecta	Hymenoptera	Formicidae	*Acromyrmex*	sp.1	X		E
Insecta	Hymenoptera	Formicidae	*Crematogaster*	sp.1	X		E
Insecta	Hymenoptera	Formicidae	*Odontomachus*	sp.1		X	E
Insecta	Hymenoptera	Formicidae	*Pachycondyla*	sp.2	X		E
Insecta	Hymenoptera	Formicidae	*Pheidole*	sp.1	X		A
Insecta	Hymenoptera	Formicidae	*Pseudomyrmex*	aff. *opaciceps*		X	A
Insecta	Isoptera	Termitidae	*Nasutitermes*	sp.	X		E/A
Insecta	Lepidoptera	Gelechioidea		sp.		X	E
Insecta	Mantodea	Mantidae		jovem		2	E
Insecta	Orthoptera	Gryllidae	Nemobiinae	sp.		1	A
Insecta	Orthoptera	Phalangopsidae	*Paraclodes*	jovem	3		E/P
Insecta	Orthoptera	Phalangopsidae	*Phalangopsis*	sp.	24	55	E/P/A
Insecta	Psocoptera	Epipsocidae	*Epipsocus*	sp.2	X	X	P
Malacostraca	Isopoda	Armadillidae		sp.1		X	E
Malacostraca	Isopoda	Philosciidae		sp.1	X	X	E/A
Amphibia	Anura	Leptodactylidae	*Leptodactylus*	cf. *vastus*	3	2	E/P
Mammalia	Chiroptera	Emballonuridae	*Peropteryx*	*kappleri*	2	2	E
Mammalia	Chiroptera	Furipteridae	*Furipterus*	*horrens*	3		P/A
Mammalia	Chiroptera	Phyllostomidae	*Trachops*	*cirrhosus*	1		P/A
